# Super-achromatic monolithic microprobe for ultrahigh-resolution endoscopic optical coherence tomography at 800 nm

**DOI:** 10.1038/s41467-017-01494-4

**Published:** 2017-11-16

**Authors:** Wu Yuan, Robert Brown, Wayne Mitzner, Lonny Yarmus, Xingde Li

**Affiliations:** 1 Department of Biomedical Engineering, School of Medicine, Johns Hopkins University, Baltimore, MD 21205 USA; 2 Department of Anesthesiology/Critical Care Medicine, School of Medicine, Johns Hopkins University, Baltimore, MD 21205 USA; 3 Department of Environmental Health Sciences, School of Medicine, Johns Hopkins University, Baltimore, MD 21205 USA; 4 Division of Pulmonary and Critical Care Medicine, School of Medicine, Johns Hopkins University, Baltimore, MD 21205 USA

## Abstract

Endoscopic optical coherence tomography (OCT) has emerged as a valuable tool for advancing our understanding of the histomorphology of various internal luminal organs and studying the pathogenesis of relevant diseases. To date, this technology affords limited resolving power for discerning subtle pathological changes associated with early diseases. In addition, it remains challenging to access small luminal organs or pass through narrow luminal sections without potentially causing trauma to tissue with a traditional OCT endoscope of a 1-1.5 mm diameter. Here we report an ultracompact (520 µm in outer diameter and 5 mm in rigid length) and super-achromatic microprobe made with a built-in monolithic fiber-optic ball lens, which achieves ultrahigh-resolution (1.7 µm axial resolution in tissue and 6 µm transverse resolution) for endoscopic OCT imaging at 800 nm. Its performance and translational potential are demonstrated by in vivo imaging of a mouse colon, a rat esophagus, and small airways in sheep.

## Abstract (web summary)

Optical coherence tomography is an established optical tool for in vivo clinical imaging and diagnosis applications, but endoscopic technologies remain limited. Here, Yuan et al. develop a flexible, narrow diameter, super-achromatic endoscopic probe for ultrahigh-resolution in vivo OCT imaging of small luminal organs or narrow constrictions.

## Introduction

Endoscopic optical coherence tomography (OCT) affords high-resolution three-dimensional (3D) in vivo visualization of internal luminal organs^1–3^. It has been demonstrated for a wide range of clinical applications such as disease diagnosis and screening where traditional biopsy suffers from sampling error or risk of complications^4–11^. However, axial resolution is limited to approximately 10 µm in air with conventional OCT operating at 1300 nm, which is suboptimal for accurately resolving fine tissue structures or subtle pathologic changes associated with early diseases. In addition, there is a practical need for a flexible catheter with a small diameter to ease its delivery and minimize potential trauma to tissue when imaging within a small luminal area (such as small airways or arteries) or passing through narrow luminal sections (such as esophageal strictures). Despite the clinical needs, a flexible OCT catheter with an ultrahigh resolution (for example, an axial resolution < 3 µm in air) and ultra-small form factor (for example, an overall outer diameter OD < 1 mm) is not yet available.

In recent years, OCT endoscopy at 800 nm has attracted increasing attention due to the unique possibility of achieving higher resolution (that is, < 3 µm versus conventional approximately 10 µm in air) and potentially better imaging contrast^12–14^. However, a broad spectrum bandwidth at 800 nm leads to severe chromatic aberration in conventional graded-index (GRIN) lens-based catheters, which results in a severely compromised axial resolution along the imaging depth (for example, with a 3–5 times worse resolution than the optimal one)^13^. Recently, our group has demonstrated that chromatic aberration can be significantly mitigated by using diffractive optics in OCT catheters^13,14^. Such catheters, however, require a customized diffractive lens and normally the catheter has an OD of about 1.3 mm. Clearly, a simpler and more cost-effective approach to control the chromatic aberration and associated degradation of axial resolution will be more attractive than using customized diffractive optics.

To minimize the size of an OCT catheter, a GRIN fiber has been used as the imaging element in the probe^12,15,16^. Further miniaturization can be achieved by using an all-fiber monolithic catheter design, which employs an angle polished GRIN fiber-tip as a beam reflector^12,15^. However, such a GRIN fiber-based catheter inherits the severe chromatic aberration at 800 nm the same as the traditional GRIN lens-based catheters. In addition, it also suffers astigmatism in the transverse beam direction. On the other hand, researchers have demonstrated that a fiber-optic ball lens can be used to achieve an anastigmatic transverse focus and a miniature catheter size similar to the case when using a GRIN fiber^17–19^. More importantly, unlike the GRIN fiber, our current study found that achromatic performance at 800 nm can be conveniently achieved by a compact design involving a fiber-optic ball lens along with a multi-mode fiber (MMF) based spacer.

In this work, we demonstrate a super-achromatic, ultracompact microprobe made of a built-in monolithic fiber-optic ball lens and beam reflector. The resultant microprobe has an overall OD of 520 µm (25-gauge) and a rigid length of 5 mm, and provides an 6 µm-focused spot size with the astigmatism ratio < 1.1. Our microprobe exhibits a super-achromatic property and produces an axial resolution of 2.4 µm in air (1.7 µm in tissue) along the imaging depth of 1.2 mm when using a home-built Ti:Sapphire laser as the OCT light source with a 3-dB spectral bandwidth of approximately 150 nm. Initially, in vivo imaging of a mouse colon is performed with the microprobe to demonstrate its performance for imaging small luminal organs. We then demonstrate its ease of delivery through a narrow luminal section by passing the probe through the upper esophageal sphincter for imaging rat esophagus in vivo. Furthermore, to demonstrate the flexibility and the translational potential of the microprobe for imaging small lumens within complex internal organs, in vivo imaging of small airways of diameter ≤ 1 mm on a sheep model is conducted with the microprobe (using bronchoscopic guidance). The benefits of the ultrahigh resolution afforded by the microprobe are evidenced by the clearly delineated fine tissue microstructures on the OCT images, which correlate well with corresponding histology.

## Results

### Monolithic super-achromatic and anastigmatic microprobe

**Fig. 1:**
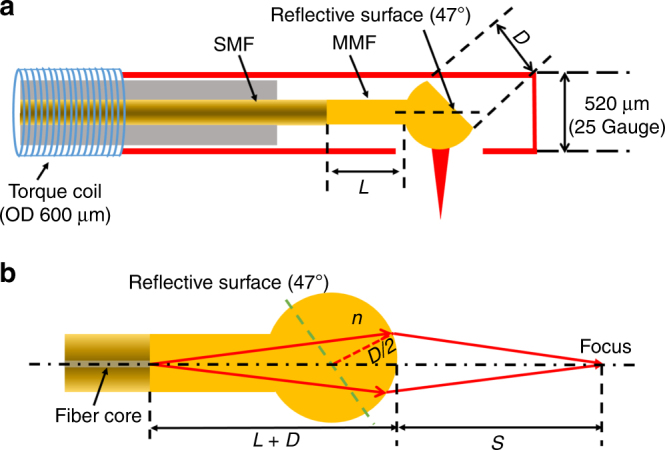
Schematic of the fiber ball lens-based microprobe. **a** The design of microprobe operating at 800 nm. SMF: single-mode fiber, MMF: multi-mode fiber, OD: outer diameter. **b** An equivalent forward-viewing design of microprobe. *L* length of the MMF, *D* diameter of the fiber ball lens, *n*: refractive index, *S* working distance

**Fig. 2:**
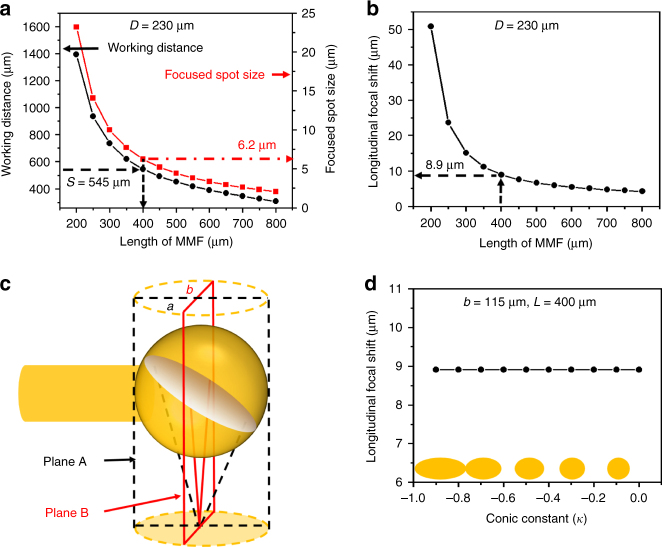
Ray-tracing simulations on the microprobe design and performance. **a** Calculated working distance and focused beam spot size versus the length of the multi-mode fiber (MMF) spacer with a fixed ball lens diameter of 230 µm. **b** Calculated longitudinal focal shift for wavelength range from 700 to 900 nm versus the length of the MMF spacer with a fixed ball lens diameter of 230 µm. **c** Schematic of the fiber ball lens with a top projected ellipse (yellow dashed line) of a major axis length *a* and a minor axis length *b*. Focused beams in the plane A that contains the major axis (black dashed lines) and the beams in the plane B that contains the minor axis (red solid lines) are illustrated, respectively. **d** Calculated longitudinal focal shift in the plane A under different conic constants when the radius of the fiber ball lens (that is, the minor axis length) is 115 µm and the MMF length is 400 µm. The representative yellow solid ellipses illustrated in the inset have a conic constant of −0.1, −0.3, −0.5, −0.7, and −0.9, respectively

Figure 1a illustrates the design schematic of our microprobe, which is simple and easy to fabricate. The microprobe consists of a home-made fiber ball lens and a short piece of MMF as a spacer (or beam expander) spliced to a single-mode fiber (SMF). The fiber ball lens was designed to have a reflective surface at 47° (between the fiber axis and silver-coated surface) in order to deflect the imaging beam for side-viewing and at the same time to avoid potential high back-reflection from the protective plastic sheath encasing the entire microprobe and the tissue surface. A 25-gauge hypodermic tube with a side opening was employed to protect the distal end, and a torque coil of a 600 µm diameter was used to protect the rest of the optical fiber and transfer rotation from the proximal to the distal end for performing circumferential beam scanning. To explicitly analyze the chromatic aberration (see “Methods” section), we simplify the microprobe optics design to its equivalent forward-viewing counterpart and assume a ball lens of a spherical shape, as shown in Fig. 1b. There are two design parameters, that is, the length of the MMF (*L*) and the diameter of the ball lens (*D*). Selecting appropriate *L* and *D* can help achieve a desired working distance (*S*). To find the conditions under which an optimal resolution and achromaticity can be achieved for a desired working distance with a given ball lens diameter, ray-tracing simulations were carried out with Zemax (Zemax, LLC.) by assuming a uniform pure silica index profile in both the beam expander and ball lens (see “Methods” section). As shown in Fig. 2a, the working distance and focused spot size were calculated versus the length of the MMF with a fixed ball lens diameter of 230 µm (see “Methods” section). It was found that a shorter working distance can be achieved with a longer MMF, and the focused spot size decreased with the increase of the MMF length. Thus, as qualitatively discussed in “Methods” section, a longer MMF is preferred. Considering a 1 mm OD protective plastic sheath (as an example) would be used together with the microprobe, a working distance of 545 µm was selected so as to have the laser beam focused at ~160 µm outside the plastic sheath. This corresponds to an MMF length of about 400 µm, at which a suitable focused spot size of 6.2 µm diameter can be achieved as highlighted in Fig. 2a. To verify the resulted chromatic aberration, further simulations of the longitudinal focal shift were performed for different MMF lengths and a fixed ball lens diameter of 230 µm. As illustrated in Fig. 2b, the longitudinal focal shift is inversely proportional to the MMF length. By choosing an 400 µm long MMF, a nearly achromatic microprobe can be achieved with only an 8.9 µm longitudinal focal shift over a broadband spectrum from 700 to 900 nm. This demonstrates in principle that a super-achromatic ultrathin microprobe with a simple design is feasible. It should also be noted that the chromatic aberration could be further reduced with a shorter working distance, which in turn depends on the OD of the plastic sheath employed for different applications.

So far, all simulations were performed under the assumption of a spherical fiber ball lens. However, in reality the shape of fiber ball lens may be elliptical. The conic constant of an ellipse is defined as $\kappa = - \left[ {\frac{{{a^2} - {b^2}}}{{{a^2}}}} \right]$, where *a* and *b* are major and minor axis lengths, respectively, as shown in Fig. 2c. It is noteworthy that the minor axis length *b* corresponds to the radius of the ball lens, that is, *D*/2 = 115 µm. It is also noted that an elliptical ball lens will introduce a focus shift for the laser beam in plane A (that is, the plane containing the major axis and enclosed in the black dashed lines) relative to the beam in plane B (that is, the plane containing the minor axis and enclosed in the red solid lines), as illustrated in Fig. 2c. On the other hand, the plastic sheath introduces a negative cylindrical lens effect, which diverges the beam in plane B while keeping the beam unchanged in plane A. To correct this, we made use of the elliptical ball lens with a slightly shorter minor axis (compared to the major axis) to provide a higher focusing power in the plane B in order to compensate the cylindrical lens effect. This basic idea has been demonstrated before^19^ and was adopted in our current design. According to our calculation with Zemax (Zemax, LLC.), when using a plastic sheath of 1 mm OD and a 120 µm wall thickness, an elliptical ball lens of *k* ≈ −0.05 (that is, *a* = 118 µm and *b* = 115 µm) can help achieve a nearly non-astigmatic design, thus achieving an optimal transverse resolution. To further study the influence of the ball lens shape on the chromatic aberration, the longitudinal focal shift in the plane A versus the conic constant was calculated with a fixed MMF length of 400 µm and a minor axis length of 115 µm. We found that the chromatic aberration was independent of the conic constant and the same longitudinal focal shift could be achieved in plane A as that in plane B (Fig. 2d). By using a slightly elliptical (or nearly spherical) ball lens, super-achromatic performance could be achieved with minimal astigmatism in our probe design.

### Characterization of the microprobe

**Fig. 3:**
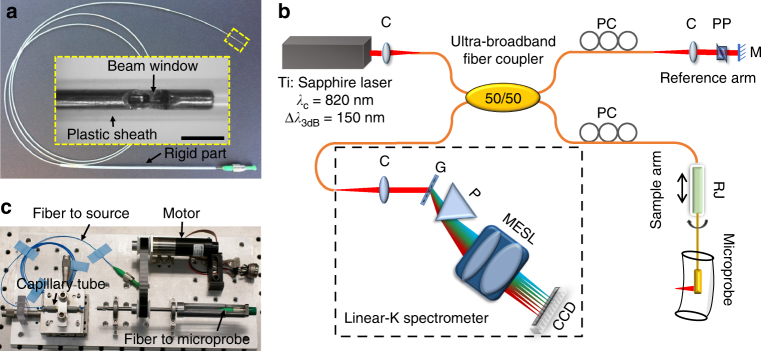
Experimental setup and microprobe prototype. **a** Photograph of the super-achromatic microprobe of 2 m in length encased within a transparent plastic sheath of a 1 mm outer diameter (OD), inset: magnified view of the microprobe distal end boxed with yellow dashed lines. Scale bar is 1 mm. **b** Schematic of the spectral-domain endoscopic optical coherence tomography (OCT) imaging system. **c** Photograph of the home-made rotary joint consisting of a glass capillary tube. C achromatic collimator, CCD line scan charge-coupled device, G grating, M mirror, MESL multi-element scan lens, P linear-K mapping prism, PC polarization controller, PP prism pair, RJ fiber-optic rotary joint

The fabrication process for the microprobe involved four major steps (see “Methods” section). The resultant all-fiber monolithic probe was encased within an 5 mm-long 25-gauge stainless steel hypodermic tube of an OD of 520 µm with a precut beam passage window (see “Methods” section). The photograph of an 2 meters long flexible microprobe encased in a transparent protective plastic sheath is shown in Fig. 3a. To characterize and demonstrate the performance of the microprobe, a spectral-domain OCT (SD-OCT) endoscopy system as shown in Fig. 3b was employed (see “Methods” section). Imaging beam rotation was performed at a speed of ~5 frames per second (fps) by using a home-made glass capillary-based rotary joint (see “Methods” section), as shown in Fig. 3c. The rotary unit was mounted on a translational stage to enable pullback during circumferential beam scanning for performing 3D imaging.

**Fig. 4:**
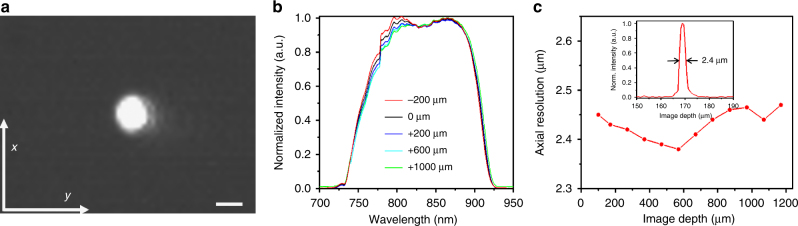
Characterization of microprobe performance. **a** Image of the focused spot captured by a charge-coupled device (CCD) camera when the microprobe was encased with a plastic sheath of a 1 mm outer diameter (OD). **b** Backreflected spectra of the microprobe by a mirror placed at different positions along the imaging depth. **c** Axial resolution measured along the imaging depth. The inset shows a representative point-spread function (PSF) of the microprobe with an 2.4 µm full-width at half-maximum (FWHM, that is, axial resolution in air). Scale bar is 5 µm

Figure 4a illustrates an image of the focused spot of the microprobe (encased with a 1 mm OD plastic sheath) captured by a CCD camera, which reveals an astigmatism ratio of < 1.1 (the ratio of the transverse resolution in the *x* direction to the one in the *y* direction). The focused spot size was measured to be 6 μm (corresponding to a measured confocal parameter of about 70 µm) with a working distance of 540 μm, which are both very close to the designed values, that is, a target lateral resolution of 6.2 μm (corresponding to a theoretical confocal parameter of about 74 µm) at a working distance of 545 μm.

Other measured performance parameters of the microprobe are also shown in Fig. 4, including the achromaticity (b) and axial resolution (c). The achromaticity of a focal imaging system (such as the microprobe) is commonly described by the longitudinal focal shift. The smaller the longitudinal focal shift, the less its influence on the OCT axial resolution. The impact of the residual longitudinal focal shift in an OCT system can be conveniently evaluated by measuring the backreflected spectra. The spectra shown in Fig. 4b were measured by placing a mirror at different locations relative to the focal plane of the microprobe along the imaging depth. The spectra were captured by an optical spectrum analyzer (OSA). It was found that the backreflected spectra remain nearly unchanged along the imaging depth, confirming that the residual longitudinal focal shift does not have much influence on the reflected spectra and thus not much influence on the OCT axial resolution. This was verified by a consistent axial resolution measured along the imaging depth, as shown in Fig. 4c, which confirmed the super-achromatic property of the microprobe. A representative point-spread function (PSF) of the microprobe (as shown in the inset of Fig. 4c) exhibited a full-width at half-maximum (FWHM) of 2.4 μm (in air) when using a home-made Ti:Sapphire laser as the light source for OCT.

### In vivo imaging of small luminal organs

**Fig. 5:**
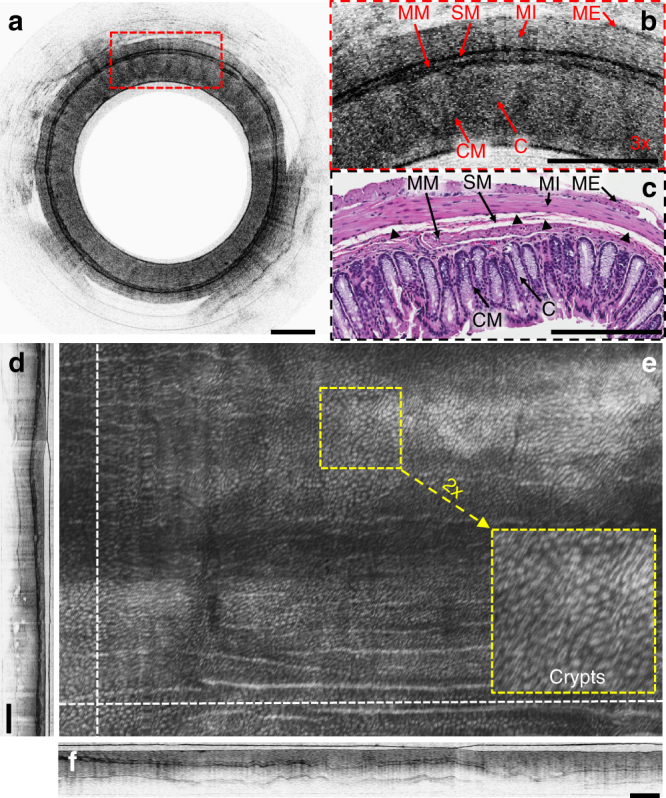
In vivo imaging of mouse colon. **a** Representative in vivo two-dimensional (2D) optical coherence tomography (OCT) image acquired by the microprobe. **b** 3x enlarged view of the area in **a**, which is boxed by red dashed lines. **c** Corresponding haemotoxylin and eosin (H&E) histology. **e** An en face projection view constructed by axial summation of 1.23 mm × 3.04 mm × 5 mm (axial × circumferential × longitudinal) field of view. Inset; 2x enlarged view of yellow dashed box. **d**, **f** Cross-sectional images along white dashed lines in **e**, which correspond to the circumferential and longitudinal direction, respectively. Tissue separations seen in the histology micrograph (labeled with black triangles in **c**) are the histology processing artifacts. All scale bars are 250 µm. C crypt, CM colonic mucosa, MI muscularis interna, ME muscularis externa, MM muscularis mucosa, SM submucosa

Our prototype microprobe featured an ultracompact form factor, super achromaticity, and ultrahigh resolution, affording an opportunity for assessment of fine structural details in small luminal organs, which can be valuable for clinical applications and facilitates its deployment to the small organs (such as infant esophagus or airway). The performance of the microprobe was first tested on imaging mouse colon in vivo. Full-circumferential images were acquired, processed, displayed, and saved in real time at about 5 fps with each frame consisting of 2048 × 8196 pixels (axial × circumferential). 3D volumetric imaging was performed by pullback of the microprobe within the stationary plastic sheath with a computerized linear translation stage at the proximal end of the probe during circumferential rotation. The pullback speed could be used to control the separation (that is, pullback pitch) between two adjacent circumferential images and 20 µm was used in our studies. A representative 2D OCT snapshot of mouse colon and its 3x enlarged view are shown in Fig. 5a, b, respectively, which demonstrate that fine tissue microstructures can be clearly resolved and correlated well with the corresponding histology (Fig. 5c). As illustrated in Fig. 5b, the layered structure of mouse colon, including the upper colonic mucosa (CM), muscularis mucosa (MM), submucosa (SM), muscularis interna (MI), and muscularis externa (ME), are clearly delineated in the ultrahigh-resolution OCT image. The crypts structure in the CM layer can also be identified, but with suboptimal contrast. This might result from tissue surface compression by the microprobe within a small lumen. As shown in Fig. 5e and the 2x zoomed-in inset, the crypts structure can be much better appreciated in an en face projection view of the 3D image (with a volume of 1.23 × 3.04 × 5 mm^3^, axial × circumferential × longitudinal) by the summation of OCT intensity along the entire imaging depth for each A-line. The mouse colonic layer structures can also be clearly visualized in the cross-sectional view along the circumferential and longitudinal direction labeled with white dashed lines in Fig. 5e, as displayed in Fig. 5d, f, respectively.

### In vivo imaging through narrow luminal sections

**Fig. 6:**
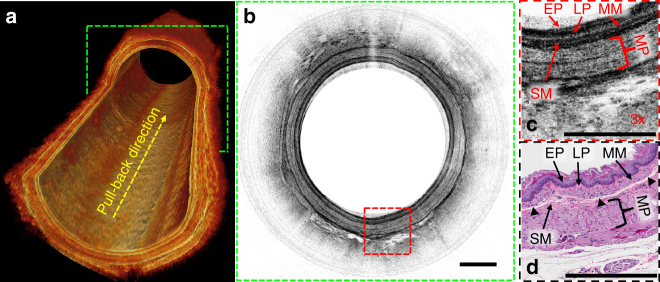
In vivo imaging of rat esophagus. **a** Cut-away view of a reconstructed three-dimensional (3D) optical coherence tomography (OCT) image representing a 4 mm-long normal rat esophagus imaged with 20 µm pullback pitch. **b** Representative in vivo two-dimensional (2D) OCT image of rat esophagus corresponding to green dashed lines boxed cross-section in (**a**). **c** 3x enlarged view of the area in **b** boxed by red dashed lines. **d** Corresponding haemotoxylin and eosin (H&E) histology. EP: keratinized stratified squamous epithelium, LP lamina propria, MM muscularis mucosae, MP: muscularis propria, SM submucosa. The histology processing artifact is labeled with black triangles in **d**. All scale bars are 250 µm

The ultracompact structure of the microprobe also facilitates passage through narrow luminal sections (such as strictures), while still affording a smooth probe rotation and pullback for 3D imaging. To demonstrate this valuable feature, in vivo imaging was performed in a rat esophagus. The microprobe was passed through the oral cavity of rat, bend towards the pharynx, bypass the trachea entrance, then advanced through the tight upper esophageal sphincter (which was used to mimic the luminal strictures where the probe delivery would face a significant resistance), and finally reach the small esophagus. Once the microprobe reached the region of interest, sequential cross-sectional images with an 6 µm × 2.4 µm resolution (lateral × axial, in air) were acquired with a 20 µm pullback pitch, and then reconstructed for 3D visualization of the layered microstructures as shown in Fig. 6a. A representative circumferential image is illustrated in Fig. 6b, corresponding to the cross-section indicated with the green dashed box in Fig. 6a. Figure 6c shows a 3x enlarged view of a representative image region indicated by the red dashed box on the cross-sectional image, where clear delineation of esophageal layered structures is evident, including the keratinized stratified squamous epithelium (EP), lamina propria (LP), muscularis mucosae (MM), submucosa (SM), muscularis propria (MP). These fine layered structures identified on the OCT image correlated well with the corresponding haemotoxylin and eosin (H&E) histology (Fig. 6d), which again confirmed the excellent resolving capability of the microprobe based 800 nm OCT technology.

### In vivo imaging of small lumens in complex internal organs

**Fig. 7:**
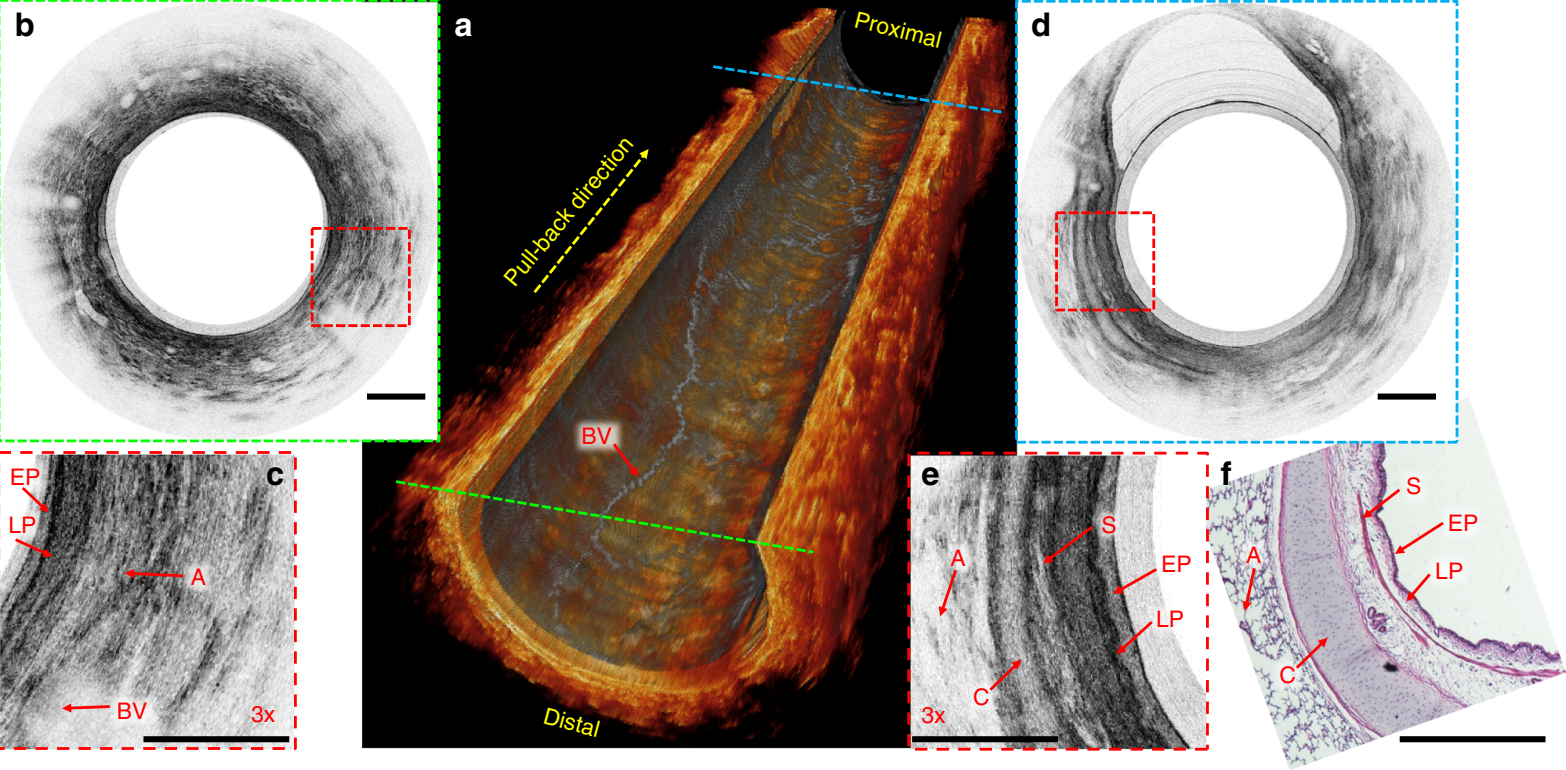
In vivo endobronchial imaging of sheep small airways. **a** Cut-away view of a reconstructed three-dimensional (3D) image of an 8 mm-long sheep small airway with the en face projection view of blood vessels overlaid on the inner surface of the three-dimensional (3D) image. **b** Representative in vivo 2D image of the peripheral sheep airway corresponding to the cross-section indicated with the green dashed line in (**a**). **c** 3x enlarged view of the area boxed with red dashed lines in (**b**). **d** Representative in vivo 2D image of the sheep small airway, which is relatively more towards the proximal side (indicated with the blue dashed line in **a**). **e** 3x enlarged view of the area boxed with red dashed line in (**d**). **f** Corresponding haemotoxylin and eosin (H&E) histology. A alveoli, BV blood vessel, C cartilage, EP epithelium, LP lamina propria, S smooth muscle. All scale bars are 250 µm

To further demonstrate the translational potential of the flexible OCT microprobe, imaging of small lumens within more complex internal organs, such as the peripheral airways (also called small airways) with a diameter of 1 mm or less, was conducted in a sheep model. It is well known that small airway wall remodeling is an important pathological feature underlying chronic obstructive pulmonary diseases (COPD)^9,11,20^. Compared with conventional imaging modalities (such as computed tomography) and conventional OCT operating at 1300 nm, in vivo endoscopic imaging of small airways with a microprobe at 800 nm provides direct 3D visualization of airway walls with ultrahigh resolution of 1.7 µm (in tissue). In our study, the microprobe was deployed distal to a 5th generation bronchus through the 2.2 mm diameter working channel of a bronchoscope, which was inserted into a 3rd generation bronchus of the right middle lung lobe. As shown in Fig. 7a, a reconstructed 3D OCT image of an 8 mm-long sheep small airway in a cut-away view clearly delineates the fine airway wall structures, which can be potentially useful for assessing pathological changes associated with COPD in small airways in vivo. In addition to fine airway wall structures, information on blood vessels can also be extracted from the same OCT volumetric dataset (with an image volume of 1.23 × 1.57 × 8 mm^3^ in axial × circumferential × longitudinal direction) by using an established algorithm called IBDV (intensity-based Doppler variance)^21^. The projection view of as-derived blood vessels (based on axial intensity summation) is also shown in Fig. 7a, which is overlaid on the inner surface of the 3D OCT structural image.

To better appreciate the fine microstructures and tissue components obtained by imaging with the microprobe in vivo, Fig. 7b shows a representative cross-sectional image, which corresponds to a distal cross-section indicated by the green dashed line in Fig. 7a. A 3x enlarged view of a representative region on the cross-sectional image indicated by the red dashed box is shown in Fig. 7c. As we can see, the characteristic structures and tissue components of the peripheral airways, including the epithelium (EP), lamina propria (LP), blood vessels (BV), and alveoli (A), can be readily identified. In addition, a representative 2D image corresponding to a proximal cross-section indicated by the blue dashed line in Fig. 7a is shown in Fig. 7d. A 3x enlarged view (Fig. 7e) of a representative area boxed with red dashed lines clearly demonstrates other important tissue components including smooth muscle (S) and cartilage (C). Again, we found that these fine microstructures and tissue components of small airways correlated well with corresponding H&E histology as shown in Fig. 7f. Such direct visualization of smooth muscle of small airways in vivo offers a unique capability and opportunity for longitudinally studying the pulmonary pathology in COPD.

## Discussion

As an emerging medical imaging technique of low risk and minimal invasiveness, conventional endoscopic OCT operating at 1300 nm was approved by the Food and Drug Administration (FDA) for imaging gastrointestinal tract (such as the esophagus) and coronary artery. Likewise, ultrahigh-resolution endoscopic OCT imaging with an ultracompact and flexible microprobe at 800 nm represents promising opportunities for in vivo assessment of tissue pathological changes in small and/or complex luminal organs, such as small airways, coronary arteries and pancreatic ducts. In addition to the improved resolution and imaging contrast over the conventional endoscopic OCT operating at 1300 nm, the reduced size and enhanced flexibility of the microprobe could potentially reduce the risk when deployed to image a small and delicate luminal organ, such as bile duct. The extremely short rigid length (5 mm) at the distal end of the probe allows for a sharp bending on the delivery path of the microprobe. The capability of obtaining ultrahigh-resolution microscopic images of a lumen over a long segment affords a better chance to detect pre-malignant or early stage cancers (before the metastasis) when curative interventions could be deployed. This opportunity is not limited to adult subjects, as the microprobe can also potentially benefit pediatric patients thanks to its ultrathin diameter. In addition, this ultrathin and super-achromatic microprobe can be potentially integrated within an excisional biopsy needle to accurately assess fine structures of the target tissue in order to guide biopsy with an improved diagnostic yield.

In principle, the form factor of the fiber ball lens-based microprobe can be further shrunk by using a thinner hypodermic tube at the distal end and/or a smaller protective plastic sheath over the entire catheter. The chromatic aberration of the microprobe can also be reduced with a shorter working distance and a longer MMF (Fig. 2b). Furthermore, the same probe design can be used to fabricate ultrathin OCT fine needle for interstitial imaging of solid tissues/organs with minimal invasiveness. It is also noted that the microprobe with a longer working distance tends to lead to a decreased lateral resolution and more importantly an increased longitudinal focal shift (Fig. 2a, b). Therefore, it is suboptimal to use a microprobe for imaging larger lumens (for example, with a diameter larger than 2 mm as suggested by Fig. 2a, b). For those cases, ultrahigh-resolution diffractive catheters with a size around 1.3 mm can be a preferable alternative^13,14^.

It is worth mentioning that fabrication of the reported microprobe only involves standard and common optical fiber handling procedures such as splicing, cleaving, polishing, and metallic coating (see “Methods” section), which can potentially be streamlined for mass production. Therefore, the as-designed microprobe can serve as a low-cost and disposable OCT catheter for translational use.

It is also worthwhile mentioning that, by following the approved imaging procedures (see “Methods” section), the potential motion artifacts (such as heart beat and breathing) can be greatly minimized during the endoscopic OCT imaging on the upper and lower gastrointestinal tracts (GI) in the small animal models and on the small airways in the ovine model, as we demonstrated in Figs. 5–7, respectively. During imaging, the small lumens with a typical diameter about 1 mm wrapped around the microprobe, which might help further suppress motion artifacts. In addition, sheep were found to be able to tolerate a short apneic episode ~1 min (by halting the ventilation system) without causing any noticeable sequela.

To conclude, a 25-gauge (520 µm) monolithic fiber-optic microprobe with a simple design and an ultracompact form factor has been developed in this work. This microprobe exhibits a super-achromatic property that enables ultrahigh-resolution (1.7 µm in tissue) OCT imaging at 800 nm with very low astigmatism. The performance of the microprobe has been systematically characterized and experimentally verified by in vivo imaging of small luminal organs, that is, mouse colon. We also demonstrated the feasibility of the microprobe to advance through narrow lumens or strictures by imaging rat esophagus in vivo. To demonstrate the flexibility and translational potential of the microprobe, in vivo bronchoscopically guided imaging of sheep small airways of a diameter 1 mm or less has also been performed. Ultrahigh-resolution microprobe-based OCT images correlated closely with corresponding histology. This low-cost high-performance microprobe provides an innovative tool for in vivo optical biopsy studies of internal luminal organs of small and/or young subjects and for easy delivery through narrow lumens or strictures.

## Methods

### Microprobe’s chromatic aberration and design considerations

As shown in Fig. 1b, the optics design of microprobe can be simplified to an equivalent forward-viewing counterpart. By using paraxial approximation and assuming a ball lens of a spherical shape, the working distance (*S*) and longitudinal focal shift ($\frac{{{\rm{d}}S}}{{{\rm{d}}\lambda }}$) can be calculated by the following two equations:

1 $$\frac{1}{S} = \frac{{n - 1}}{{D/2}} - \frac{n}{{L + D}}$$

2 $$\frac{{{\rm{d}}S}}{{{\rm{d}}\lambda }} = - \left( {\frac{2}{D} - \frac{1}{{L + D}}} \right) \cdot {S^2} \cdot \frac{{{\rm{d}}n}}{{{\rm{d}}\lambda }},$$

where *n* is the refractive index, *λ* is the wavelength, and $\frac{{{\rm{d}}n}}{{{\rm{d}}\lambda }}$ is the material dispersion.

As seen from Eqs. 1 and 2, several considerations should be taken to achieve an achromatic design. First, a short working distance is desirable for a small longitudinal focal shift. Second, for a given working distance, Eq. 2 can be combined with Eq. 1 and rewritten as $\frac{{{\rm{d}}S}}{{{\rm{d}}\lambda }} = - \left( {\frac{1}{S} + \frac{2}{D}} \right) \cdot \frac{1}{n} \cdot {S^2} \cdot \frac{{{\rm{d}}n}}{{{\rm{d}}\lambda }}$, which indicates that the longitudinal focal shift decreases with the increase of the ball lens diameter. Third, for a given ball lens diameter, a longer MMF length *L* will lead to a shorter working distance *S*, and thus a smaller longitudinal focal shift. Finally, a low material dispersion is highly desirable for achieving low achromatic aberration in the microprobe.

On the basis of the aforementioned considerations, an MMF (105/125 µm, core/cladding diameter) with a pure silica core was chosen due to: First, its relatively low refractive index and dispersion at 800 nm (compared with commonly used doped silica core); second, a close match between the simulations (by assuming a uniform pure silica index profile) and the experimental results (with the MMF) confirmed that the thin cladding of MMF has negligible impact on the ball lens performance (Supplementary Table 1 and Supplementary Note 1). In order to achieve the smallest chromatic aberration for a given working distance, the fiber ball lens should have a diameter as large as possible. For the prototype microprobe with a target size of about 270 µm (that is, the inner diameter of the 25-gauge protective hypodermic tube), a fiber ball lens diameter of 230 µm was chosen with a sufficient reserved space between the fiber ball lens and the inner wall of the hypodermic tube for easy encasing. Once the ball lens diameter and the MMF material were decided, the optimal longitudinal focal shift was then determined (or tuned) by the MMF length.

### The microprobe fabrication procedures

**Fig. 8:**
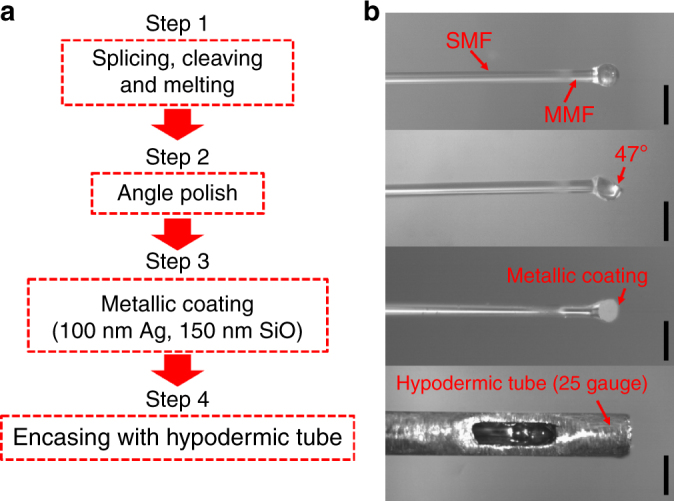
Fabrication procedures. **a** Flow chart for microprobe fabrication. **b** Representative photographs of the fiber probe corresponding to each fabrication step shown in **a**. All scale bars are 500 µm

There are four major steps (Fig. 8a) involved in the fabrication process for our microprobe. As shown in Fig. 8b, the MMF (FG105LVA, Thorlabs Inc.) was first fusion-spliced to the SMF (HI780, Corning Inc.), and then precisely cleaved to a predefined length under a microscope. The fiber ball lens was formed at the distal end of the MMF by using an electric arc fusion splicer (FSU 995FA, Ericsson). The parameters of the fusion splicer (that is, the fusion time, the fusion current, and fusion discharge times, see details in Supplementary Note 2) were finely tuned to achieve a ball lens with a desired diameter and a nearly spherical shape. In general, we found that, with the same fusion current, a shorter fusion time leads to an elliptical shape, and a longer fusion time results in a near spherical shape.

The second step was to polish a flat surface out of the ball lens with a tilting angle of 47°, as displayed in Fig. 8b. The third step was to apply a 100 nm-thick silver coating on the polished surface with another 150 nm protective layer of silicon monoxide on the top of the silver to achieve a reflectivity of 87%. In contrast to the use of total internal reflection (TIR, requiring an incident angle on the polished surface beyond the critical angle of about 43.5°) for beam deflection, the protected silver coating can prevent beam from leaking through the polished surface and help achieve excellent reflectivity over a broad incident angle (that is, below the TIR angular range) and a uniform focused spot as well.

The last step encased the all-fiber monolithic probe within an 5 mm-long stainless-steel hypodermic tube (25 gauge, OD 520 µm) with a precut beam passage window. UV curable glue was used to fill the gap between the fiber ball lens and the hypodermic tube, as displayed in Fig. 8b. A torque coil (Asahi Intecc Co.) of a 600/330 µm in outer/inner diameter was then used to encase the rest of the optical fiber. Finally, a transparent plastic sheath of a 1 mm OD and a 120 µm wall thickness was used to protect the entire microprobe during imaging.

### Ultrahigh-resolution 800 nm SD-OCT endoscopy system

An SD-OCT endoscopy system (Fig. 3b) was built to characterize and demonstrate the performance of the microprobe. It consists of a home-made Ti:Sapphire laser (with a center wavelength around 820 nm and a 3-dB bandwidth of ~150 nm), an ultra-broadband fiber coupler, and a home-built linear-k spectrometer of 2048 pixels, which can accommodate an about 250 nm spectral bandwidth centered around 800 nm and offer an imaging depth of 1.2 mm^13^. A detection sensitivity of about −108 dB (for an incident beam power of 7 mW in the sample arm) and a near-theoretical-limit sensitivity roll-off of about −13 dB mm^−1^ have been achieved. A home-made glass capillary-based rotary joint (Fig. 3c) was used to perform imaging beam rotation. Such a rotary joint has been previously demonstrated and its performance was further optimized to achieve one-way throughput >91%, optical coupling variation less than ± 5% during continuous rotation, and measured back-reflection < −58 dB^13,14^.

### Animal studies

In vivo animal imaging protocols, including mouse colon, rat esophagus, and sheep airways imaging, were all approved by the Institutional Review Board of the Johns Hopkins University. Mice and rats were under anesthesia (with 100 mg kg^−1^ ketamine and 16 mg kg^−1^ xylazine) during OCT imaging, where the microprobe was directly deployed to the lower colon sections (near the rectosigmoid junction) and the upper esophagus sections (right beyond the upper esophageal sphincter), respectively. The sheep anesthesia was initiated with ketamine (25 mg kg^−1^) and then maintained by continuous intravascular (IV) infusion of propofol (800 μg kg^−1^ h^−1^) during imaging. The sheep trachea was intubated and the lung was mechanically ventilated at 14 breaths per minute with a tidal volume adequate to obtain an end tidal carbon dioxide of approximately 30 mm Hg. Bronchoscope (Olympus BF-p40) was deployed to the sheep airways of the right middle lobe. During OCT imaging, the ventilator was stopped for about 1 min (with a maximum permitted halt time of 2 min) with vital signs continuously monitored. Immediately after each imaging session, ventilation was resumed. We found the animals tolerated these short apneic episodes with no noticeable sequela. It is worth mentioning that breath hold (or halt of ventilation) is a well-established clinic procedure where a duration of 1–2 min for anesthetized patients was not uncommon. After OCT imaging, the animals were sacrificed and imaged tissues were harvested and placed in formalin overnight before submitted for histological processing. Standard H&E histological slides were obtained and correlated with the endoscopic OCT images.

### Data availability

The data that support the findings of this study are available from the corresponding author upon request.

## Electronic supplementary material

Supplementary Information
