# Author Correction: Super-achromatic monolithic microprobe for ultrahigh-resolution endoscopic optical coherence tomography at 800 nm

**DOI:** 10.1038/s41467-018-03637-7

**Published:** 2018-03-14

**Authors:** Wu Yuan, Robert Brown, Wayne Mitzner, Lonny Yarmus, Xingde Li

**Affiliations:** 1 Department of Biomedical Engineering, School of Medicine, Johns Hopkins University, Baltimore, MD 21205 USA; 2 Department of Anesthesiology/Critical Care Medicine, School of Medicine Johns Hopkins University, Baltimore, MD 21205 USA; 3 Department of Environmental Health Sciences, School of Medicine, Johns Hopkins University, Baltimore, MD 21205 USA; 4 Division of Pulmonary and Critical Care Medicine, School of Medicine, Johns Hopkins University, Baltimore, MD 21205 USA

Correction to: *Nature Communications* 10.1038/s41467-017-01494-4; published online 16 November 2017.

The original version of this Article contained an error in the third sentence of the ‘Animal studies’ section of the Methods, which incorrectly read ‘The sheep anesthesia was initiated with ketamine (25 mg kg^−1^) and then maintained by continuous intravascular (IV) infusion of propofol (800 mg kg^−1^ h^−1^) during imaging.’ The correct version states ‘800 µg kg^−1^ h^−1^’ in place of ‘800 mg kg^−1^ h^−1^’. This has been corrected in both the PDF and HTML versions of the article.

