# Supplementary material for: Super-achromatic monolithic microprobe for ultrahigh-resolution endoscopic optical coherence tomography at 800 nm
Source: Nat Commun. 2017 Nov 16;8:1531. doi: 10.1038/s41467-017-01494-4 (PMC5688175; doi:10.1038/s41467-017-01494-4)
Supplement: Supplementary file 1 — Supplementary Information [file 41467_2017_1494_MOESM1_ESM.pdf]

Supplementary Table 1. Comparison of the measured performance parameters of a ball lens made of a multi-mode fiber (MMF) with theoretical predictions for a ball lens made of a pure silica coreless fiber.

Ball lens	Lens diameter	MMF length	Working distance (theory/measurement)	Focused spot size (theory/measurement)
#1*	230 μm	400 μm	545/540 μm	6.2/6.0 μm
#2	230 μm	500 μm	451/455 μm	4.4/4.5 μm
#3	230 μm	350 μm	619/620 μm	7.7/8.0 μm
#4	248 μm	400 μm	639/635 μm	7.1/7.0 μm
#5	216 μm	400 μm	462/460 μm	5.7/6.0 μm

*The reported microprobe.

Supplementary Note 1: Comparison of simulations with experimental results for the microprobe

Considering the cladding of the multi-mode fiber (MMF) is thin (10 um) and the refractive index of the cladding is very close to the core (i.e. 1.450 for cladding versus 1.453 for the pure silica core at 820 nm), the impact of the cladding on the ball lens is expected to be small if not negligible.

In order to confirm the potential impact of the MMF cladding on the ball lens performance, we have fabricated several microprobes and carried out a series of simulations and measurements. The simulation (or theoretical) results for a ball lens made of a coreless fiber (with a uniform pure silica index profile in both the beam expander and ball lens) and the measured parameters for a ball lens made of an MMF are shown in Supplementary Table 1 above. As we can see, a close match between the theoretical predictions (with a coreless fiber) and the experimental results (with an MMF) confirmed that the thin cladding has negligible impact on the ball lens performance.

Supplementary Note 2: Customized parameters of the fusion splicer for ball lens fabrication

A fusion procedure was customized with the fusion splicer (FSU 995FA, Ericsson) for fabricating the ball lens. The key fusion steps and representative empirical parameters used for making ball lens of 230 μm in diameter are: first step, prefusion for 0.3 seconds with a current of 10 mA; second step, first fusion discharge for 10 seconds with a current of 21 mA; third step, second fusion discharge for 1 second with a current of 1 mA; and last step: third fusion discharge for 2 seconds with a current of 1 mA.
